# The role of autophagy in Parkinson's disease: rotenone-based modeling

**DOI:** 10.1186/1744-9081-9-13

**Published:** 2013-03-15

**Authors:** Nian Xiong, Jing Xiong, Min Jia, Ling Liu, Xiaowei Zhang, Zhenzhen Chen, Jinsha Huang, Zhentao Zhang, Lingling Hou, Zhijian Luo, Devina Ghoorah, Zhicheng Lin, Tao Wang

**Affiliations:** 1 Department of Neurology, Union Hospital, Tongji Medical College, Huazhong University of Science and Technology, 1277 Jiefang Road, Wuhan, Hubei, 430022, China; 2 Department of Neurology, Renmin Hospital of Wuhan University, Wuhan, 430060, China; 3 Department of Neurology, The First Hospital of Jingzhou, Clinical Medical College, Yangtze University, Jingzhou, 434000, China; 4 Department of Radiology, Union Hospital, Tongji Medical College, Huazhong University of Science and Technology, Wuhan, Hubei, China; 5 Department of Psychiatry, Harvard Medical School; Laboratory of Psychiatric Neurogenomics, Division of Alcohol and Drug Abuse, and Mailman Neuroscience Research Center, McLean Hospital, Belmont, MA, USA; 6 Harvard NeuroDiscovery Center, Boston, MA, 02114, USA

**Keywords:** Autophagy, LC3, Parkinson's Desease, Rotenone, Autophagosome, Pathogenesis

## Abstract

**Background:**

Autophagy-mediated self-digestion of cytoplasmic inclusions may be protective against neurodegenerative diseases such as Parkinson’s disease (PD). However, excessive autophagic activation evokes autophagic programmed cell death.

**Methods:**

In this study, we aimed at exploring the role of autophagy in the pathogenesis of rotenone-induced cellular and animal models for PD.

**Results:**

Reactive oxygen species over-generation, mitochondrial membrane potential reduction or apoptosis rate elevation occurred in a dose-dependent fashion in rotenone-treated human neuroblastoma cell line SH-SY5Y. The time- and dose-dependent increases in autophagic marker microtubule-associated protein1 light chain 3 (LC3) expression and decreases in autophagic adaptor protein P62 were observed in this cellular model. LC3-positive autophagic vacuoles were colocalized with alpha-synuclein-overexpressed aggregations. Moreover, the number of autophagic vacuoles was increased in rotenone-based PD models *in vitro* and *in vivo*.

**Conclusions:**

These data, along with our previous finding showing rotenone-induced toxicity was prevented by the autophagy enhancers and was aggravated by the autophagy inhibitors in SH-SY5Y, suggest that autophagy contributes to the pathogenesis of PD, attenuates the rotenone toxicity and possibly represents a new subcellular target for treating PD.

## Background

Parkinson’s Disease (PD) is the second most common neurodegenerative disease and affects as many as 1–2 % of the worldwide population aging at 60 years and older [1]. The pathological hallmarks of PD include the loss of dopaminergic (DA) neurons in the substantia nigra pars compacta (SNc) and DA terminals in the striatum, and the presence of proteinaceous cytoplasmic inclusions called Lewy bodies. Currently, the precise pathogenic mechanisms in PD remain incompletely understood. As Lewy Bodies contain aggregated alpha-synuclein, ubiquitin and other misfolded proteins [1], a growing attention has been drawn to the role of autophagy in the pathogenesis of PD [2]. Different from ubiquitin-proteasome system which degrades proteins through the narrow barrel of proteasome, macroautophagy (referred to below as autophagy) is responsible for the largely non-specific bulk degradation of long-lived cytosolic proteins and organelles.

It is acknowledged that autophagy is related to PD. Autophagosomes containing neuromelanin and lipofuscin have been identified in degenerating neurons in brains with PD [3]. Moreover, overexpressions of alpha-synuclein mutants have been reported to activate autophagy [4-6]. Rapamycin (Rap), known as an autophagy inducer, is neuroprotective in parkinsonian cellular and mice models, by enhancing autophagy to degrade misfolded proteins [7]. Our previous findings suggested that valproic acid and carbamazepine (the most commonly used anti-epilepsy and mood-stabilizing medications with low-risk and easy administration), as well as Rap and lithium, might be potential therapeutics for PD as autophagy enhancers [8]. On the other hand, 3-methyladenine, an autophagy inhibitor, has reportedly offered neuroprotection against 6-hydroxydopamine toxicity [9], suggesting that excessive activation of autophagy during neuronal loss participates in the pathogenesis pathway of PD [10]. That is to say, both autophagy inducer and inhibitor have been proven to serve as neuroprotectors against PD. There are still many controversial and unsolved problems regarding the role of autophagy in PD. First, whether it is autophagy activation or autophagy suppression that confers neuroprotection against PD; second, whether autophagy is a defense mechanism or a response to the DA neuron death; third, whether autophagy is a key mechanism or just an innocent bystander in the pathogenesis of PD [11,12]. Therefore, we need to better understand the role of autophagy in the pathogenesis of PD prior to any clinical application of autophagy-based medications in PD subjects.

Rotenone, a potent mitochondrial complex I inhibitor, is one of the most relevant neurotoxins to induce parkinsonian symptoms [6,13-15]. Despite debates, the rotenone model is able to recapitulate slow and specific loss of DA neurons and over-expression of alpha-synuclein and better mimics the clinical features of idiopathic PD [16-20]. Among the various models for PD, the rotenone model has recently drawn particular attention for two reasons: 1) it reproduces most of the motor symptoms and the histopathological features of PD, including Lewy bodies [21,22]; and 2) rotenone and other pesticides are powerful inhibitors of mitochondrial respiration and associated with the higher incidence of sporadic Parkinsonism among the population of rural areas [23-26]. Thus, rotenone-induced parkinsonian models were chosen to explore the role of autophagy in PD in this study. We found that rotenone induced time- and dose-dependent apoptosis of SH-SY5Y cells increases the autophagic marker microtubule-associated protein1 light chain 3 expression, and increases the number of autophagic vacuoles, and decreases the autophagic adaptor protein P62 expression. These data indicated that autophagy was involved in the pathogenesis of rotenone-induced PD models, revealing a neuroprotective alternative to treating PD.

## Methods

### Cell culture

SH-SY5Y cells (American type culture collection, gift from Dr. Jianguo Chen) were cultured in DMEM/F12 medium (Invitrogen, Carlsbad, CA, USA) supplemented with 10% fetal bovine serum (Invitrogen) at 37°C with 5% CO_2_ and 95% air (vol/vol). Rotenone (Sigma-Aldrich, St. louis, MO, USA) was dissolved in dimethyl sulfoxide before dilution with the culture medium. The final concentration of dimethyl sulfoxide (DMSO) per well was 0.2%. DMSO alone was added to the culture medium in control group (“Con-group”). For the dose-dependent study, rotenone was given at a concentration of 0.1, 0.5, 1, 2.5, 5, 10 and 20 μM for 24 hours. For the time-dependent study, rotenone (2.5μM) was given for 3, 6, 12, 24, 36 or 48 hours to induce cell damage.

### MTT assay

Cell viability was assessed by the 3-(4,5-Dimethylthiazol-2-yl)-2,5-diphenyltetrazolium bromide (MTT) method [27,28]. The MTT assay is a colorimetric assay of the activity of cellular enzymes that reduce the tetrazolium dye, MTT, into insoluble formazan, giving a purple color. Briefly, SH-SY5Y cells were plated at a density of 1 × 10^4^ cells per well in 96-well plates. After exposure to rotenone and vehicle, 20 μl of MTT (5 mg/ml, Sigma-Aldrich) was added into each well before incubation in a humidified incubator at 37°C for 4 hours to allow the formation of purple formazan crystal. Then, 100 μl of the solubilization reagent (0.1 N HCl in anhydrous isopropanol, Sigma-Aldrich) was added into each well and lysate spectrophotometrically measured for absorption at λ 570 nm with background subtraction at 690 nm. Cell viability was expressed as a percentage of the value in untreated control cells.

### Detection of apoptosis, mitochondrial membrane potential (MMP) and reactive oxygen species (ROS) in SH-SY5Y cells

Annexin V was used to probe phosphatidylserine expression on the cell surface, an event found in apoptosis as well as other forms of cell death [29]. In this study, staurosporine (50nM, Sigma-Aldrich) treatment for 24 hours was employed as a positive control to induce cell apoptosis. SH-SY5Y cells were harvested after treatment with 0.25% trypsin, washed with phosphate buffered solution (PBS) and incubated in PBS containing the Annexin V-fluorescein isothiocyanate (Annexin V, 5 μl in 100 μl PBS) and Propidium Iodide (PI, 100 μg/ml working solution, 1 μl in 100 μl) at 37°C in darkness for 15 minutes. The apoptosis rate = [Annexin V(+)PI(−) cells + Annexin V(+)PI(+) cells] /total cell × 100%. The specific fluorescence of 10,000 cells was analyzed on FACScalibur (BD Biosciences, Franklin Lakes, NJ, USA) within 1 hour after antigen antibody reaction [27-29]. Data were analyzed by using FSC express version 3.0 (De Novo Software, Los Angeles, CA, USA).

It was reported that a decrease in MMP was one of the earliest events in apoptosis [30]. When stained with JC-1, red fluorescence of mitochondria was due to the formation of J-aggregates at high MMP, and green fluorescence of mitochondria to the formation of JC-1 monomers at low MMP [31]. ROS detection was based on ROS-catalyzed formation of fluorescent compound DCF. The nonfluorescent probe DCFH-DA could diffuse passively through the cellular membrane. With intracellular esterase activity, DCFH-DA formed a nonfluorescent compound DCFH, which was oxidized into the fluorescent compound DCF by ROS [32]. For the analysis of MMP and ROS, cells were harvested, resuspended in PBS and immediately stained with JC-1 (1 mg/ml in DMSO, Molecular Probes, Eugene, OR, USA) [31] or DCFH-DA (10 μM, Invitrogen) [32], and incubated at 37°C for 30 minutes in the darkness. After washing with ice-cold PBS twice, the samples were subject to FACScan flow Cytometry. Data were analyzed again by using FSC express version 3.0 (De Novo Software).

### Immunoblotting

The microtubule-associated protein1 light chain 3 (LC3) was a marker for all types of autophagic vacuolar organelles. A higher LC3 expression level meant more autophagic vacuolar organelles in the cells. It was reported that LC3 expression level could be related to the induction of autophagy or a block of autophagy and subsequent accumulation of LC3 [33-35]. The mammalian proteins p62 and NBR1 were selectively degraded by autophagy and could act as cargo receptors or adaptors for the autophagic degradation of ubiquitinated substrates [36,37]. The conversion of LC3-I into LC3-II (LC3-II level compared to LC3-I level) and the expression of P62 (which was degraded by autophagy) were indicative of autophagic activity. Higher ratio of LC3-II/LC3-I and lower p62 expression means higher autophagic activity. In this study, LC3 and p62 levels were measured by an immunoblotting method [27,28,38]. Cells were rinsed twice with cold PBS and lysed in buffer (50 mM Tris–HCl, pH 7.5, 100 mM NaCl, 1% NP-40, 0.5% sodium deoxycholate, 0.1% SDS,1 mM EDTA, 1 mM sodium orthovanadate,10 mM sodium fluoride, and 100 mg/ml PMSF). After incubation on ice for 30 minutes, cell lysates were then clarified by centrifugation at 12,000 × *g* and 4°C for 10 minutes and the supernatant saved for protein analysis and Western blotting. Total protein concentration was determined by the BCA kit (Sigma-Aldrich). Equal amounts of proteins (30 μg) were fractionated by 15% SDS-PAGE, and transferred to nitrocellulose membrane. The membrane was blocked with 5% non-fat milk in Tris-buffered saline (TBS) for 1 hour at room temperature, followed by incubation with primary antibodies against LC3, P62 (Sigma-Aldrich, St. Louis, MO, USA) and β-actin (Santa Cruz, Santa Cruz, CA, USA) overnight at 4°C. The membranes were then washed twice with TBS tween-20 and probed with the corresponding secondary antibodies conjugated with HRP at room temperature for 1 hour. Detection was carried out using an enhanced chemiluminescence detection kit (Pierce, Rockford, IL, USA), followed by autoradiography. The relative intensity of bands was quantified using Quantity One analysis system (Quantity One, Hercules, CA, USA). All data from three independent experiments were expressed as the ratio to optical density values of the corresponding controls for statistical analyses.

### Immunostaining

SH-SY5Y cells grown on cover slips were fixed with 4% paraformaldehyde at 4°C for 30 minutes, washed with PBS and permeabilized with 0.1% Triton-X100 and 5% bovine serum albumin (Invitrogen) in PBS [38], followed by incubation at 4°C overnight with the LC3 antibody (1:100, rabbit polyclonal antibody, Sigma-Aldrich, St. Louis, MO, USA) without or with alpha-synuclein (SNCA) antibody (1:100, mouse monoclonal antibody, Billerica, MA, USA). The corresponding secondary FITC-conjugated donkey-anti-rabbit IgG (1:200, vol/vol, Proteintech, Chicago, IL, USA) without or with Cy3-conjugated goat-anti-mouse IgG (1:500, vol/vol, Proteintech, Chicago, IL, USA) diluted in 10 μg/ml Hoechst 33258 (Sigma-Aldrich) was applied at room temperature for 1 hour. Cells were observed by using a confocal microscope (Olympas, Tokyo, Japan) and the images (50 per group, repeat for 3 times) were analyzed by a design-based unbiased method and a morphometry/image analysis system (Image-Pro Plus 6.0 software package, Bethesda, MD, USA; Edit-Convert to-Gray Scale- Enhance-Invert contrast-Apply contrast –Measure-Count/Size-Measure-Density Mean).

### Rotenone-induced hemiparkinsonian rat model

This study was approved by the Ethical Committee on Animal Experimentation of Tongji Medical College, Huazhong University of Science and Technology, China. The rotenone-induced stereotaxical hemiparkinsonian rat (inbred adult female Sprague–Dawley rats, 220–260 g) model was used in this study [6]. Briefly, animals were anesthetized with chloral hydrate (400 mg/kg in 0.9% NaCl, i.p.) and fastened on a cotton bed over a stereotaxic frame (RWD Life Science, Shenzhen, China). Rotenone dissolved in DMSO (3 μg/μl) was infused into the right ventral tegmental area (AP: 5.0 mm; L: 1.0 mm; DV: 7.8 mm) at a flow rate of 0.2 μl/minute. The needle was left in place for additional 5 minutes for complete diffusion of the drug. Rotenone was infused into the right SNc (AP: 5.0 mm; L: 2.0 mm; DV: 8.0 mm) at a flow rate of 0.2 μl/minute, with a 5-minute needle retention. After needle withdrawal, proper postoperative care was given until the animals recovered completely. The animals were administrated with ibuprofen and penicillin in the drinking water for 24 hours to alleviate potential postsurgical discomfort and to prevent infection.

### Ultrastructural study

The preparation for electron microscopy (EM) was described previously [6]. Harvested by detaching with 0.25% trypsin, SH-SY5Y were washed twice in PBS, and then fixed in 0.01 M PBS containing 2.5% glutaraldehyde. For the brain tissues, the animals were sacrificed 1 day, 2 days, 1 week, 2 weeks or 4 weeks after the stereological surgery. A 1-mm^3^ tissue block from the left and right SNc regions (−4.5 to −6.2 mm caudal to the bregma) was micro-punched, fixed in PBS containing 2.5% glutaraldehyde, and preserved at 4°C for further processing. The fragments were post-fixed in 1% osmium tetroxide in the same buffer, dehydrated in graded alcohols, embedded in Epon 812, sectioned with an ultramicrotome, and stained with uranyl acetate and lead citrate. The sections were examined with a transmission electron microscope (TEM; Technai 10, Philips, the Netherlands). For the SH-SY5Y cell-based study, the ultrastructural images were quantified by randomly counting of 100 cells and assessing the percentage of the cells with one or more autophagic vacuoles [39]. For the animals study, three blocks of SNc from each group were sectioned discontinuously for 5 slices, and 50 cell profiles (with a nucleus) were examined on each grid for counting the autophagic vacuoles per cell profile at X 13,500 magnification [40,41]. Each experiment was conducted in triplicate.

### Statistical analyses

Statistical analysis was carried out by using SPSS version 12.0 for Windows software (SPSS, Chicago, IL, USA). Given a normal distribution in all groups, intergroup differences were assessed by one-way analysis of variance (ANOVA) followed by Least square difference's post hoc test [42]. Results are presented as mean ± SEM, with *P* value of < 0.05 as statistically significant.

## Results

### Rotenone affected SH-SY5Y cell proliferation time- and dose-dependently

**Figure 1:**
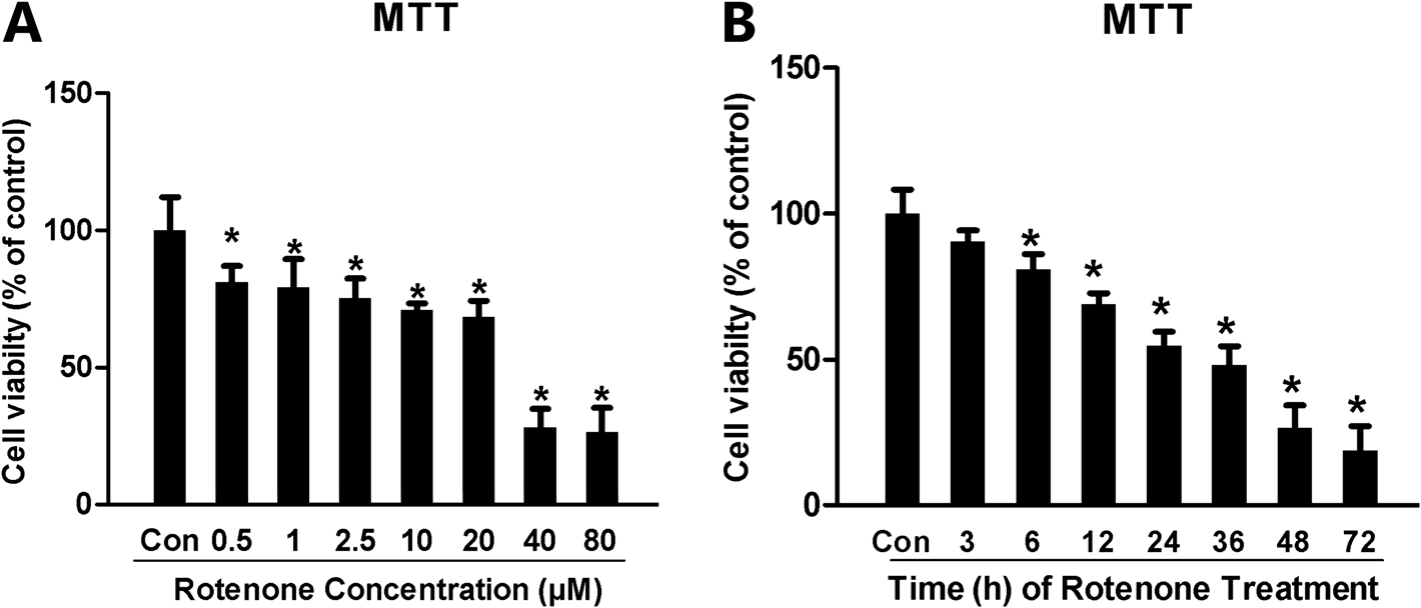
**Rotenone dose- and time-dependently induced cytotoxicity in SH-SY5Y.** The MTT assay was employed to assess the cell viability after rotenone administration. For dose-dependence, rotenone was given at a concentration of 0.1, 0.5, 1, 2.5, 5, 10 and 20 μM for 24 hours. For time-dependence, rotenone (2.5μM) was given for 3, 6, 12, 24, 36 and 48 hours respectively to induce cell damage. (**A**) Dose-dependent effect of 24-hour treatment with rotenone on cell proliferation in SH-SY5Y (relative MTT value); (**B**) Time-dependent effect of rotenone (2.5 μM) on SH-SY5Y viability with different experimental time points (from 1–48 hours after rotenone administration; *P < 0.05, compared to Con-group).

We first assessed the effects of different concentrations of rotenone on the proliferation of SH-SY5Y cells based on the MTT test. Compared with Con-group, rotenone administration for 24 hours caused a significant decrease in cell proliferation in 0.5, 1, 2.5, 10, 20, 40 and 80 μM group, respectively (Figure 1A). For the time-dependence study, rotenone (2.5μM) significantly decreased the relative MTT value by 19.01%, 30.88%, 45.04%, 51.86%, 73.40% and 81.1% in Rot-3h, Rot-6h, Rot-12h, Rot-24h, Rot-36h , Rot-48h and Rot-72h group compared with the Con-group (Figure 1B).

### Rotenone induced dose- and time-dependent apoptosis, MMP reduction and ROS generation in cultured SH-SY5Y cells

**Figure 2:**
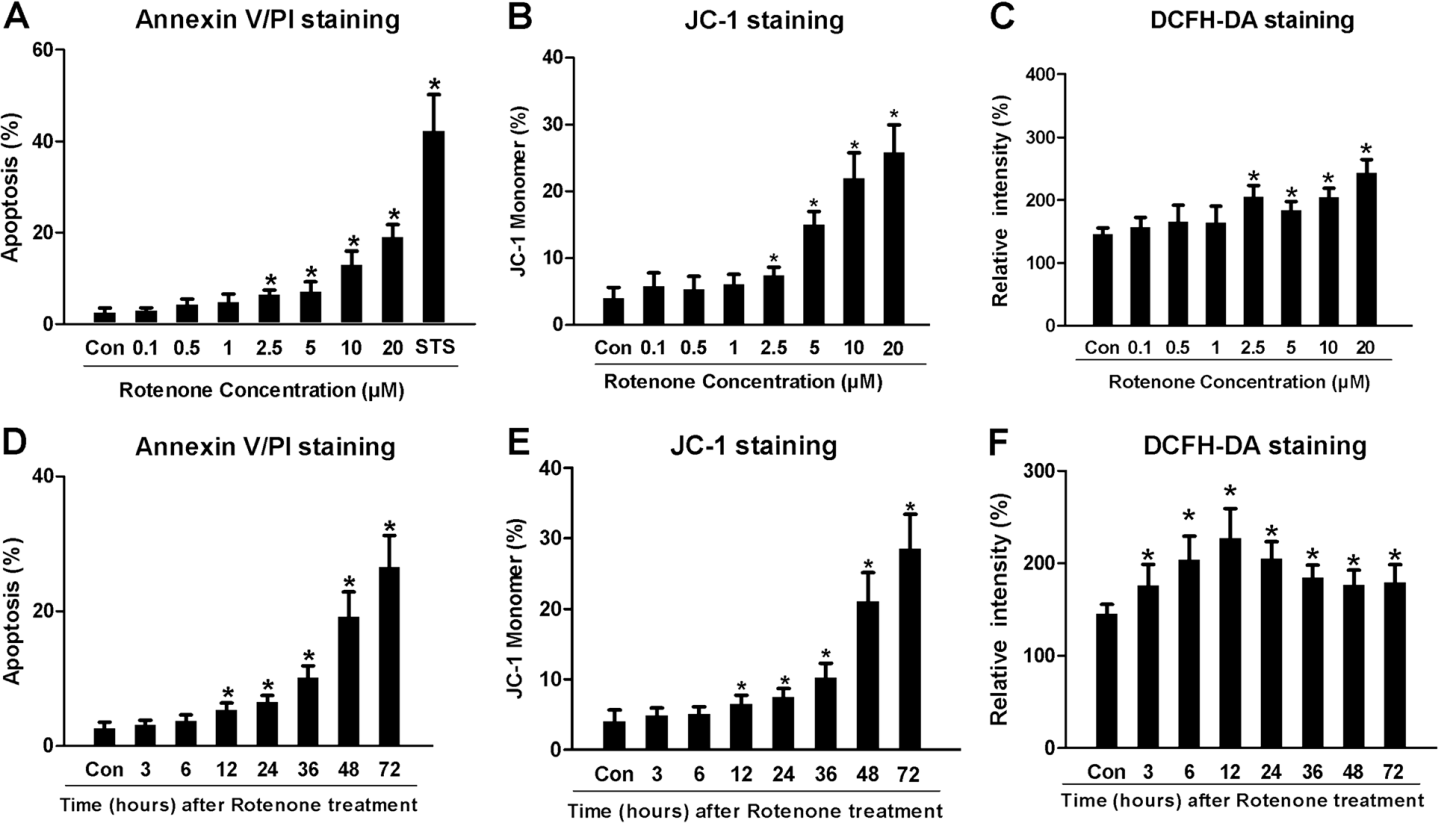
**Rotenone evoked apoptosis, MMP reduction and ROS generation dose- and time-dependently in SH-SY5Y.** For Annexin V/PI double-staining, the apoptosis rate was a sum of both early and late apoptosis rates (apoptosis rate = [Annexin V(+)PI(−) cells + Annexin V(+)PI(+) cells] /total cell × 100%), staurosporine (STS, 50nM, Sigma-Aldrich) treatment for 24 hours was used as a positive control to induce cell apoptosis. The apoptosis rate of Con-, Rot-0.1μM, Rot-0.5μM, Rot-1μM, Rot-2.5μM, Rot-5μM, Rot-10μM, Rot-20μM and STS-group was 2.60±0.90%, 3.00±0.60%, 4.30±1.20%, 4.80±1.80%, 6.50±0.98%, 7.20±2.10%, 13.00±3.00%, 19.00±2.70% and 42.31±7.90%, respectively. The JC-1 staining was used to assess the MMP of SH-SY5Y cells, the percentages of JC-1 monomers presented the percentages of SH-SY5Y cells with low MMP. The relative intensity of DCF was employed to evaluate the ROS level in SH-SY5Y cells. (**A**), (**D**): Statistical analysis of apoptosis rates in different groups. (**B**), (**E**): Relative percentage of JC-1 monomers-positive cells (cells with low MMP) in different groups. (**C**), (**F**): Fluorescence intensity of DCFH in all groups. (*P < 0.05, compared to Con-group).

It is unclear whether the decreased MTT value was attributable to rotenone-based inhibition of cell proliferation or rotenone-induced increases of SH-SY5Y apoptosis. To clarify the effects of rotenone on SH-SY5Y cells, we further performed the apoptosis assessment by Annexin V/PI double-staining and JC-1 staining. In order to detect the effects of rotenone on apoptosis, SH-SY5Y cells were double-stained with FITC-conjugated Annexin V and PI. The dose-dependent data indicated that rotenone treatment evoked concentration-dependent apoptosis in SH-SY5Y cells. The apoptosis rate of the Rot-2.5 μM , Rot-5μM, Rot-10μM, Rot-20μM or staurosporine group was statistically significantly different from that in Con-group (Figure 2A). As the amount of the shift from J-aggregates to JC-1 monomer, 4.02±1.62%, 5.79±2.04%, 5.43±1.86%, 6.12±1.45%, 7.48±1.20%, 15.02±1.95%, 21.94±3.83% and 25.84±4.15% of SH-SY5Y cells formatted JC-1 monomers in Con-, Rot-0.1μM, Rot-0.5μM, Rot-1μM, Rot-2.5μM, Rot-5μM, Rot-10μM and Rot-20μM group, respectively (Figure 2B). Rotenone infusion caused ROS generation in Rot-0.1μM, Rot-0.5μM, Rot-1μM, Rot-2.5μM, Rot-5μM, Rot-10μM or Rot-20μM group compared with Con-group (Figure 2C). The ROS generation in the Rot-2.5μM, Rot-5μM, Rot-10μM or Rot-20μM group was significantly different from that in the Con-group. Rotenone (2.5 μM) conspicuously evoked the apoptosis (Figure 2D) and MMP reduction (Figure 2E) of SH-SY5Y cells in a time-dependent fashion as well. After 12 hours treatment with 2.5 μM rotenone, SH-SY5Y cells began to show apoptotic changes and MMP reduction. Moreover, 3-hour treatment with rotenone (2.5 μM) caused time-dependently significant ROS generation and the ROS reached the crest value from the 12-hour to 72 hour time points in SH-SY5Y cells (Figure 2F).

### Rotenone up-regulated LC3 expression and down-regulated P62 expression in SH-SY5Y at an early stage after administration

**Figure 3:**
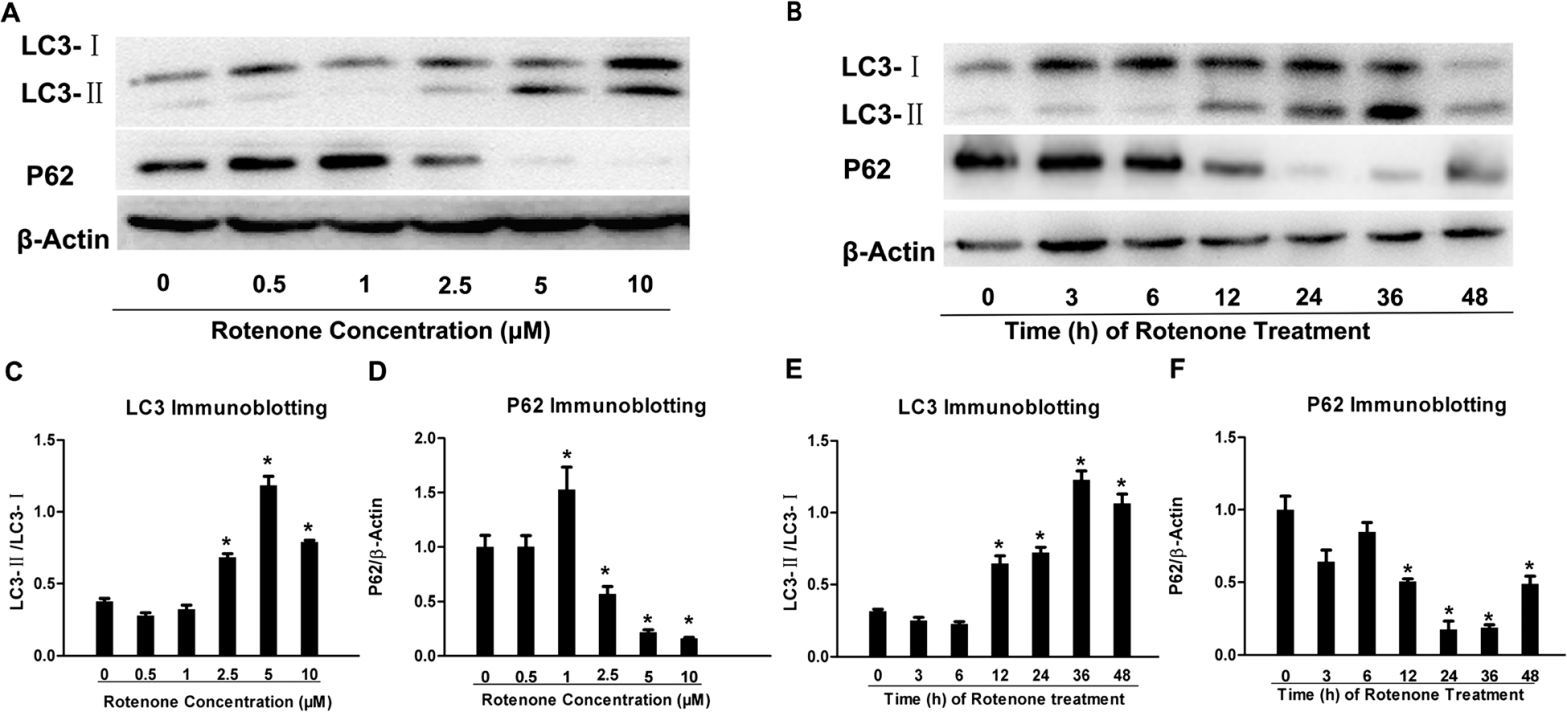
**Rotenone activated autophagy in SH-SY5Y.** LC3/P62 immunoblotting was used to track the conversion of LC3-I into LC3-II and the expression of P62 for autophagic activity. (**A**) The immunoblotting of LC3-II/LC3-I, P62 and β-actin from Con-, Rot-0.5μM, Rot-1μM, Rot-2.5μM, Rot-5μM and Rot-10μM groups; (**C**), (**D**) Quantitative analysis of immunoblotting of LC3-II/LC3-I and P62 both controlled by β-actin, respectively. (**B**) The immunoblotting of LC3- II/I, P62 and β-actin in a Rot (2.5μM) time-dependent manner from Con-, Rot-3h, Rot-6h, Rot-12h, Rot-24h, Rot-36h and Rot-48h groups; (**E**), (**F**) Quantitative analysis of immunoblotting of LC3-II/LC3-I, P62 controlled by β-actin (*P < 0.05, compared to Con-group).

**Figure 4:**
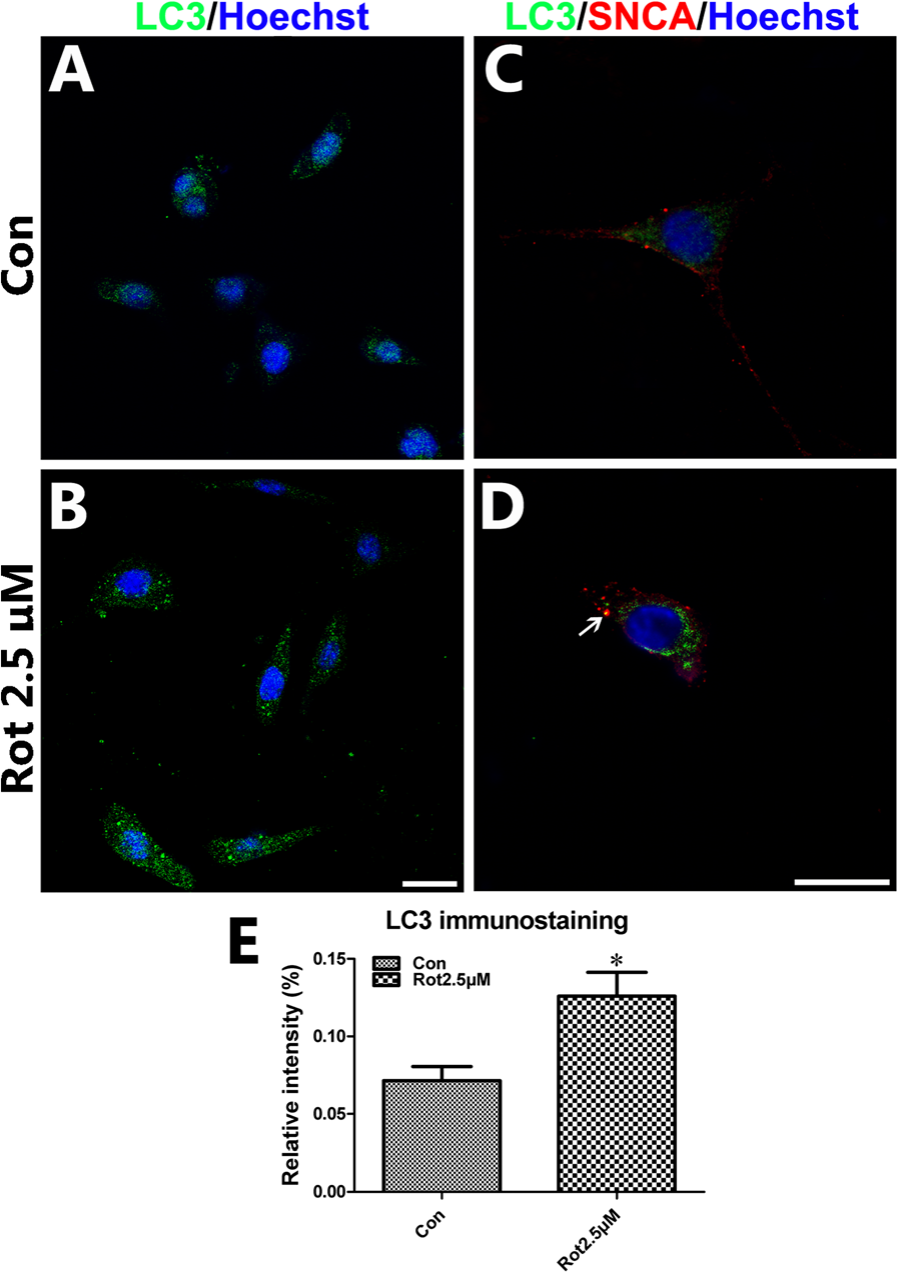
**LC3 immunostaining in rotenone-treated SH-SY5Y.** Immunostaining was performed to assess the LC3/SNCA expression and distribution in SH-SY5Y cells treated with or without rotenone (2.5μM for 24 hours). The nuclei were stained by Hoechst33258. (**A**, **B**) LC3 immunofluorescence staining for Con- and Rot-2.5μM group; (**C**, **D**) LC3/SNCA double immunostaining for Con- and Rot-2.5μM group; (**E**) statistical analysis of relative mean fluorescence intensity of LC3 immunofluorescence staining (Scale bar = 20 μm; *P < 0.05, compared to Con-group).

The Western blotting study showed that the ratio of LC3-II to LC3- I in Rot-2.5μM, Rot-5μM or Rot-10μM group was 80.20%, 212.48% or 108.55% higher than that in Con-group. There was no significant difference between Rot-0.5μM orRot-1μM and Con-groups (Figure 3A, C). The expression of P62 in Rot-2.5μM, Rot-5μM and Rot-10μM group was significant lower than the Con-group. The P62 expression in Rot-1μM, Rot-2.5μM, Rot-5μM or Rot-10μM was obviously different from that in the Con-group (Figure 3A, D). To confirm the LC3 expression and to observe the LC3 distribution in cells, the LC3 immunostaining was employed. The relative mean fluorescence intensity of LC3 was significant higher in the Rot-2.5μM compared to that in the Con-group (Figure 4A,B and E). LC3/SNCA double-immunostaining showed that SNCA-overexpressed aggregations were colocalized with LC3-positive autophagic vacuoles (Figure 4C, D), demonstrating that autophagy was involved in abnormal protein degradation in the rotenone-induced cell model of PD.

Data from the time-dependence study indicated the ratio of LC3-II to LC3- I in Rot-12h, Rot-24h, Rot-36h and Rot-48h group was significant higher than that in Con-group. There was no significant difference between Rot-3h, Rot-6h and Con-groups (Figure 3B, E). The expression of P62 in Rot-3h, Rot-12h, Rot-24h, Rot-36h and Rot-48h group was 23.48%, 58.82%, 90.02%, 68.32% and 93.02% lower than control group (Figure 3B, F). These data indicated the activation of autophagy pathway in SH-SY5Y cells at the early stage after rotenone infusion (within 24 hours after rotenone treatment).

### Rotenone induced autophagic vacuole formation in SH-SY5Y cells and SNc neurons

**Figure 5:**
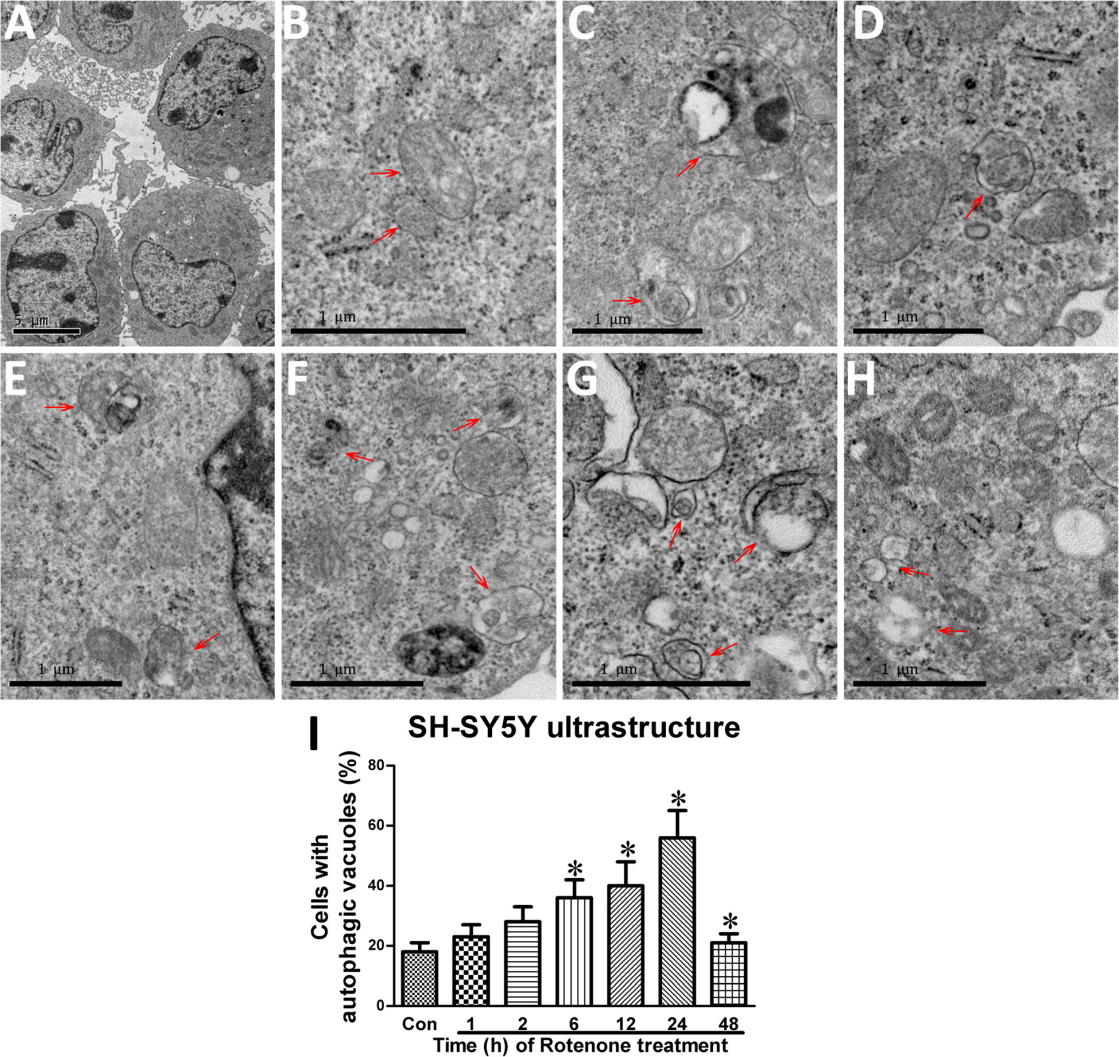
**Rotenone-induced ultrastructural changes in SH-SY5Y. The ultrastructural changes in different rotenone-treated groups.A**: high nucleo-cytoplasmic ratio and large nucleolus of SH-SY5Y cells of Con-group; **B**: normal mitochondria, rough endoplasmic reticulum and ribosomes (Con-group); **C**: mitochondria, rough endoplasmic reticulum, ribosomes and autophagic vacuoles (Arrow, Rot-1h group); **D**: mitochondria, rough endoplasmic reticulum, ribosomes and autophagic vacuoles (Arrow, Rot-2h group); **E**: rough endoplasmic reticulum, ribosomes and autophagic vacuoles (Arrow, Rot-6h group); **F**: mitochondrial swelling, mitochondrial crest fracture, mitochondrial vacuolar degeneration and autophagic vacuoles (Arrow, Rot-12h group); **G**: mitochondrial swelling, mitochondrial crest fracture, mitochondrial vacuolar degeneration and autophagic vacuoles (Arrow, Rot-24h group); **H**: mitochondrial swelling, mitochondrial crest fracture, mitochondrial vacuolar degeneration (Arrow, Rot-48h group); **I**: quantitative analysis of the rate of autophagic vacuoles-containing cells (*P < 0.05, compared to Con-group).

The ultrastructural manifestation represents a “gold standard” method to identify autophagic vacuoles [43]. Observed through a TEM, SH-SY5Y had a high nucleo-cytoplasmic ratio and a large nucleus, an irregular appearance (Figure 5A). In the absence of rotenone, there were no autophagic vacuoles except normal mitochondria, rough endoplasmic reticulum, and ribosomes which were observed in SH-SY5Y cells (Figure 5B). In the presence of rotenone, there was time-dependent formation of autophagic vacuoles, mitochondrial swelling, mitochondrial crest fracture and mitochondrial vacuolar degeneration within 24-hour administration (Figure 5C-I). Interestingly, an increase in mitochondrial numbers could be observed.

**Figure 6:**
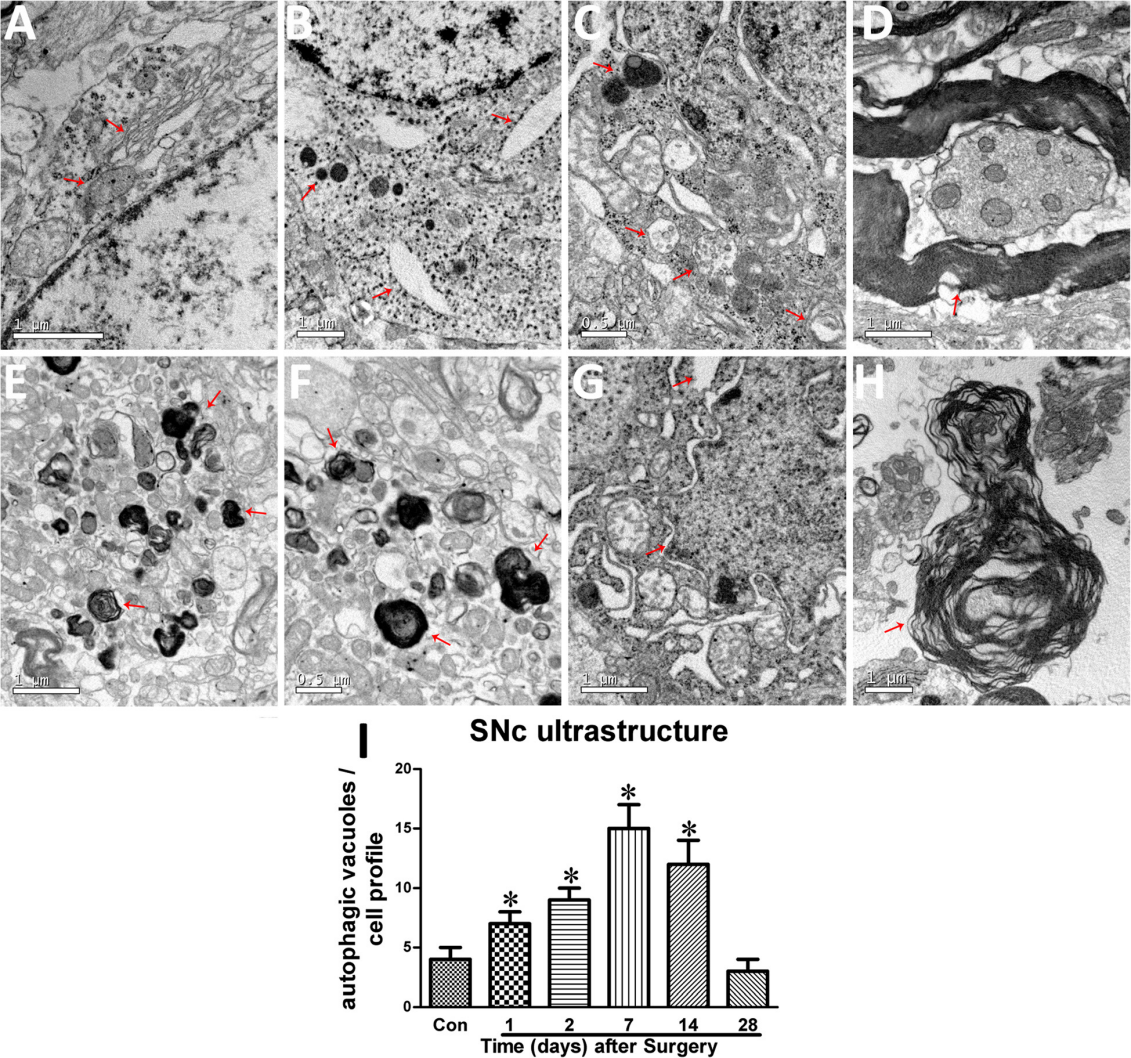
**Rotenone-induced ultrastructural changes in SNc of parkinsonian rats. A**: normal mitochondria, Golgi complex and ribosomes in the contralateral SNc of the parkinsonian animals; **B** and **C**: mitochondrial swelling, mitochondrial crest fracture, mitochondrial vacuolar degeneration, dilated and broken rough endoplasmic reticula, increase in autophagic vacuoles and increase in lysosome density in lesioned SNc of the parkinsonian animals one or two days after surgery; **D**: normal medullary sheathes in the contralateral SNc of the parkinsonian animals; **E**, **F** and **G**: mitochondrial swelling, mitochondrial crest fracture, mitochondrial vacuolar degeneration, dilated and broken rough endoplasmic reticula, lipofuscin deposition, perinuclear space augmentation and/or increase in autophagic vacuoles in lesioned SNc of the parkinsonian animals one, two or four weeks after surgery; **H**: degeneration of the medullary sheathes in lesioned SNc of the parkinsonian animals four weeks after surgery; **I**: quantitative analysis of autophagic vacuoles/unit area in SNc neurons (see Table 1 for frequency of occurrence of these ultrastructural changes at various time points; *P < 0.05, compared to Con-group).

Our previous study developed a rotenone (the mitochondrial complex-I inhibitor) model by stereotaxical infusion with small doses of rotenone into two brain sites: the right ventral tegmental area and the substantia nigra. As a result, this small dose infusion decreased tyrosine hydroxylase (TH) immunoreactivity in the infusion side by 43.7% four weeks after the infusion [6]. The rotenone infusion also reduced the DA content, the glutathione and superoxide dismutase activities, and induced alpha-synuclein expression, comparing to the contralateral side. This ST model displayed neither peripheral toxicity nor mortality, had a high success rate, and recapitulated the slow and specific loss of DA neurons [6]. Here, the left and right SNc (from 4.5 to 6.2 mm caudal to the bregma) of parkinsonian animals were micropunched and examined by using a TEM. Normal mitochondria, Golgi complex, ribosomes and medullary sheathes (Figure 6A), and normal medullary sheathes (Figure 6D) could be observed in the contralateral SNc of the parkinsonian animals. By contrast, there were mitochondrial swelling, mitochondrial crest fracture, mitochondrial vacuolar degeneration, dilated and broken rough endoplasmic reticula, lipofuscin deposition, perinuclear space augmentation, degeneration of medullary sheathes, increase in autophagic vacuoles and lysosomes in the lesioned SNc of the parkinsonian animals (Figure 6B-C, E-I, and Table 1).

**Table 1:** Rotenone-induced ultrastructural changes in SNc (semi-quantification)

	**Normal**	**Mitochondrial swelling**	**Mitochondrial crest fracture**	**Mitochondrial vacuolar degeneration**	**Dilated and broken rough endoplasmic reticula**	**Lipofuscin deposition**	**Perinular space augmentation**	**increase of autophagic vacuoles/lysosomes**	**Degeneration of medullary sheathes**
Contralateral SNc (Figure 6A)	Mitochondrial complex ribosomes, medullary sheathes								
One day after surgery (Figure 6B)		+	+	+	+			+	
Two day after surgery (6C)		++	++	++	++			++	
One week after surgery (Figure6E)		+++	+++	+++		++			
Two weeks after surgery (6F)		+++	+++	+++		++			
Four weeks after surgery (Figure 6G,H)		+++	+++	+++	++		++	+	++

Rotenone-administrated animals were sacrificed 1 day, 2 days, 1 week, 2 weeks or 4 weeks after the stereological surgery. A 1-mm3 tissue block from the ipsilateral and contralateral SNc regions (−4.5 to −6.2 mm caudal to the bregma) was micro-punched for TEM assessment. “+”, frequency of rotenone-induced ultrastructural changes in SNc, including mitochondrial swelling, mitochondrial crest fracture, mitochondrial vacuolar degeneration, dilated and broken rough endoplasmic reticula, lipofuscin deposition, perinuclear space augmentation and increase in autophagic vacuoles.

## Discussion

In this study, we have shown that 1) rotenone induces dose- and time-dependent apoptosis, MMP reduction and ROS generation in SH-SY5Y; 2) rotenone causes time- or dose-dependent upregulation of LC3 expression and decrease in P62 expression in SH-SY5Y; 3) rotenone induces autophagic vacuole formation in SH-SY5Y cells and SNc neurons and 4) LC3-positive autophagic vacuoles are colocalized with SNCA-overexpressed aggregations. These data demonstrate the involvement of autophagy in rotenone-induced parkinsonian models both *in vitro* and *in vivo*.

We suggest that autophagy activation offers neuroprotection against rotenone-caused parkinsonian. In this study, the data showed a time-dependent activation of autophagy at the first 36 hours after rotenone administration and a dramatic decrease in autophagy level in these cells 48 hours after rotenone treatment. Autophagy is an important cellular response to stress like toxins and oxidative stress. The accumulation of autophagic vacuoles in the cytoplasm of SH-SY5Y cells may be attributable to rotenone-induced toxicity via oxidative stress and mitochondrial dysfunction. These findings are similar to the results from previous studies which showing that oxidative stress may be upstream process of autophagy [44]. The activation of autophagy may help to prevent cell damage as a compensatory auto-regulative mechanism. However, when the overload of pathogenic stress exceeds cellular compensation capability, the autophagy may be under the ability to maintain the cellular balance and ultimately lead to cell death [45]. The neuroprotective effects of Rap and the neurotoxic effects of Chl on these models further confirm that the autophagy enhancement is protective [8].

Previous studies have shown that both autophagy inhibition and enhancement are neuroprotective [7,9,46,47]. The difference may be attributed to different models, different mechanisms involved in these models and different treatment phases. Increasing evidence demonstrates that early-stage activation of autophagy is protective and late-stage over activation of autophagy eventually leads to cell death. Late-stage neuronal cell loss generally occurs via autophagy [48]. Abnormal manipulation of autophagy can result in autophagic cell death or protein-aggregated neurodegeneration [49-52]. Therefore, precise autophagy regulation rather than massive autophagy enhancement or inhibition should be a therapeutic direction of PD.

We suggest that autophagy is a key mechanism involved in DA cell death rather than an innocent bystander for following reasons. 1) An increase in autophagy-related structures has been found in parkinsonian patients [53] and models, suggesting autophagy is involved; 2) pretreatment of SH-SY5Y cells with the autophagy enhancer Rap is neuroprotective while pretreatment of SH-SY5Y cells with the autophagy inhibitor Chl is toxic [8]; and 3) genetically selective manipulation of autophagy-related genes causes neurodegeneration and behavioral deficits in animals [49-51,54].

## Conclusions

Autophagy is involved in the pathogenesis of rotenone-induced PD; autophagy enhancement provides a potential therapeutic alternative for PD. Moreover, autophagy is a defense mechanism responsive to rotenone stress for the DA cell death. Enhancement of autophagy confers neuroprotection against rotenone toxicity. However, which autophagy enhancer, such as Rap, lithium, valproic acid, carbamazepine or trehalose, would be the most suitable one for PD patients remains unknown. Further endeavors are needed to address how to maintain proper cellular autophagy level, as well as the safety issues regarding long-term application of autophagy-related drugs to PD subjects.

## Abbreviations

PD: Parkinson's disease; LC3: Microtubule-associated protein1 light chain 3; DA: Dopaminergic; SNc: Substantia nigra pars compacta; Rap: Rapamycin; DMSO: Dimethyl sulfoxide; Con-group: Control group; MTT: 3-(4,5-Dimethylthiazol-2-yl)-2,5-diphenyltetrazolium bromide; PBS: Phosphate buffered solution; MMP: Mitochondrial membrane potential; ROS: Reactive oxygen species; PI: Propidium Iodide; Annexin V: Annexin V-fluorescein isothiocyanate; TBS: Tris buffered saline; SNCA: Alpha-synuclein; EM: Electron microscopy; TEM: Transmission electron microscope.

## Competing interest

There are no actual or potential conflicts of interest.

## Authors’ contributions

NX, JX, MJ, LL, XZ, ZC, JH, ZJL, ZTZ, ZCL, TW contributed to the conception and design. NX, JX, MJ, ZC, JH, LH, ZJL, HY, ZTZ took care of the cell culture studies. NX, JX, MJ, ZCL, ZTZ analyzed and interoperated the data. NX, JX, MJ, LL, DG, WT, ZCL coordinated all the experiments and helped to draft the manuscript. All authors read, revised and approved the final manuscript.

## References

[B1] Olanow CW Stern MB Sethi K The scientific and clinical basis for the treatment of Parkinson disease (2009) Neurology 2009 72 21 Suppl 4 S1 136 19470958 10.1212/WNL.0b013e3181a1d44c

[B2] Verhoef LG Lindsten K Masucci MG Dantuma NP Aggregate formation inhibits proteasomal degradation of polyglutamine proteins Hum Mol Genet 2002 11 22 2689 2700 10.1093/hmg/11.22.2689 12374759

[B3] Xilouri M Vogiatzi T Vekrellis K Stefanis L alpha-synuclein degradation by autophagic pathways: a potential key to Parkinson's disease pathogenesis Autophagy 2008 4 7 917 919 18708765 10.4161/auto.6685

[B4] Cuervo AM Stefanis L Fredenburg R Lansbury PT Sulzer D Impaired degradation of mutant alpha-synuclein by chaperone-mediated autophagy Science 2004 305 5688 1292 1295 10.1126/science.1101738 15333840

[B5] Vogiatzi T Xilouri M Vekrellis K Stefanis L Wild type alpha-synuclein is degraded by chaperone-mediated autophagy and macroautophagy in neuronal cells J Biol Chem 2008 283 35 23542 23556 10.1074/jbc.M801992200 18566453 PMC2527094

[B6] Xiong N Huang J Zhang Z Xiong J Liu X Jia M Wang F Chen C Cao X Liang Z Stereotaxical infusion of rotenone: a reliable rodent model for Parkinson's disease PLoS One 2009 4 11 e7878 10.1371/journal.pone.0007878 19924288 PMC2774159

[B7] Pan T Kondo S Zhu W Xie W Jankovic J Le W Neuroprotection of rapamycin in lactacystin-induced neurodegeneration via autophagy enhancement Neurobiol Dis 2008 32 1 16 25 10.1016/j.nbd.2008.06.003 18640276

[B8] Xiong N Jia M Chen C Xiong J Zhang Z Huang J Hou L Yang H Cao X Liang Z Potential autophagy enhancers attenuate rotenone-induced toxicity in SH-SY5Y Neuroscience 2011 199 292 302 22056603 10.1016/j.neuroscience.2011.10.031

[B9] Li L Wang X Fei X Xia L Qin Z Liang Z Parkinson's disease involves autophagy and abnormal distribution of cathepsin L Neurosci Lett 2011 489 1 62 67 10.1016/j.neulet.2010.11.068 21134415

[B10] Bredesen DE Rao RV Mehlen P Cell death in the nervous system Nature 2006 443 7113 796 802 10.1038/nature05293 17051206 PMC3970704

[B11] Cheung ZH Ip NY The emerging role of autophagy in Parkinson's disease Mol Brain 2009 2 1 29 10.1186/1756-6606-2-29 19754977 PMC2754442

[B12] Rami A Review: autophagy in neurodegeneration: firefighter and/or incendiarist? Neuropathol Appl Neurobiol 2009 35 5 449 461 10.1111/j.1365-2990.2009.01034.x 19555462

[B13] Betarbet R Sherer TB MacKenzie G Garcia-Osuna M Panov AV Greenamyre JT Chronic systemic pesticide exposure reproduces features of Parkinson's disease Nat Neurosci 2000 3 12 1301 1306 10.1038/81834 11100151

[B14] Feng Y Liang ZH Wang T Qiao X Liu HJ Sun SG: alpha-Synuclein redistributed and aggregated in rotenone-induced Parkinson's disease rats Neurosci Bull 2006 22 5 288 293 17690729

[B15] Xiong N Long X Xiong J Jia M Chen C Huang J Ghoorah D Kong X Lin Z Wang T Mitochondrial complex I inhibitor rotenone-induced toxicity and its potential mechanisms in Parkinson's disease models Crit Rev Toxicol 2012 42 7 613 632 10.3109/10408444.2012.680431 22574684

[B16] Xiong N Zhang Z Huang J Chen C Jia M Xiong J Liu X Wang F Cao X Liang Z VEGF-expressing human umbilical cord mesenchymal stem cells, an improved therapy strategy for Parkinson's disease Gene Ther 2011 18 4 394 402 10.1038/gt.2010.152 21107440

[B17] Xiong N Cao X Zhang Z Huang J Chen C Jia M Xiong J Liang Z Sun S Lin Z Long-term efficacy and safety of human umbilical cord mesenchymal stromal cells in rotenone-induced hemiparkinsonian rats Biol Blood Marrow Transplant 2010 16 11 1519 1529 10.1016/j.bbmt.2010.06.004 20542126

[B18] Cannon JR Tapias V Na HM Honick AS Drolet RE Greenamyre JT A highly reproducible rotenone model of Parkinson's disease Neurobiol Dis 2009 34 2 279 290 10.1016/j.nbd.2009.01.016 19385059 PMC2757935

[B19] Pan-Montojo F Anichtchik O Dening Y Knels L Pursche S Jung R Jackson S Gille G Spillantini MG Reichmann H Progression of Parkinson's disease pathology is reproduced by intragastric administration of rotenone in mice PLoS One 2010 5 1 e8762 10.1371/journal.pone.0008762 20098733 PMC2808242

[B20] Greenamyre JT Cannon JR Drolet R Mastroberardino PG Lessons from the rotenone model of Parkinson's disease Trends Pharmacol Sci 2010 31 4 141 142 author reply 142–143. -10.1016/j.tips.2009.12.006 20096940 PMC2846992

[B21] Betarbet R Sherer TB Greenamyre JT Animal models of Parkinson's disease Bioessays 2002 24 4 308 318 10.1002/bies.10067 11948617

[B22] Schober A Classic toxin-induced animal models of Parkinson's disease: 6-OHDA and MPTP Cell Tissue Res 2004 318 1 215 224 10.1007/s00441-004-0938-y 15503155

[B23] Landrigan PJ Sonawane B Butler RN Trasande L Callan R Droller D Early environmental origins of neurodegenerative disease in later life Environ Health Perspect 2005 113 9 1230 1233 10.1289/ehp.7571 16140633 PMC1280407

[B24] Priyadarshi A Khuder SA Schaub EA Priyadarshi SS Environmental risk factors and Parkinson's disease: a metaanalysis Environ Res 2001 86 2 122 127 10.1006/enrs.2001.4264 11437458

[B25] Dhillon AS Tarbutton GL Levin JL Plotkin GM Lowry LK Nalbone JT Shepherd S Pesticide/environmental exposures and Parkinson's disease in East Texas J Agromedicine 2008 13 1 37 48 10.1080/10599240801986215 19042691

[B26] Tanner CM Kamel F Ross GW Hoppin JA Goldman SM Korell M Marras C Bhudhikanok GS Kasten M Chade AR Rotenone, paraquat, and Parkinson's disease Environ Health Perspect 2011 119 6 866 872 10.1289/ehp.1002839 21269927 PMC3114824

[B27] Ma R Xiong N Huang C Tang Q Hu B Xiang J Li G Erythropoietin protects PC12 cells from beta-amyloid(25–35)-induced apoptosis via PI3K/Akt signaling pathway Neuropharmacology 2009 56 6–7 1027 1034 19268480 10.1016/j.neuropharm.2009.02.006

[B28] Zhang Z Cao X Xiong N Wang H Huang J Sun S Liang Z Wang T DNA polymerase-beta is required for 1-methyl-4-phenylpyridinium-induced apoptotic death in neurons Apoptosis 2010 15 1 105 115 10.1007/s10495-009-0425-8 19937276

[B29] Koopman G Reutelingsperger CP Kuijten GA Keehnen RM Pals ST van Oers MH Annexin V for flow cytometric detection of phosphatidylserine expression on B cells undergoing apoptosis Blood 1994 84 5 1415 1420 8068938

[B30] Wadia JS Chalmers-Redman RM Ju WJ Carlile GW Phillips JL Fraser AD Tatton WG Mitochondrial membrane potential and nuclear changes in apoptosis caused by serum and nerve growth factor withdrawal: time course and modification by (−)-deprenyl J Neurosci 1998 18 3 932 947 9437015 10.1523/JNEUROSCI.18-03-00932.1998 PMC6792769

[B31] Salvioli S Ardizzoni A Franceschi C Cossarizza A JC-1, but not DiOC6(3) or rhodamine 123, is a reliable fluorescent probe to assess delta psi changes in intact cells: implications for studies on mitochondrial functionality during apoptosis FEBS Lett 1997 411 1 77 82 10.1016/S0014-5793(97)00669-8 9247146

[B32] Giardino I Edelstein D Brownlee M BCL-2 expression or antioxidants prevent hyperglycemia-induced formation of intracellular advanced glycation endproducts in bovine endothelial cells J Clin Invest 1996 97 6 1422 1428 10.1172/JCI118563 8617874 PMC507201

[B33] Cherra SJ 3rd Kulich SM Uechi G Balasubramani M Mountzouris J Day BW Chu CT Regulation of the autophagy protein LC3 by phosphorylation J Cell Biol 2010 190 4 533 539 10.1083/jcb.201002108 20713600 PMC2928022

[B34] Kouroku Y Fujita E Tanida I Ueno T Isoai A Kumagai H Ogawa S Kaufman RJ Kominami E Momoi T ER stress (PERK/eIF2alpha phosphorylation) mediates the polyglutamine-induced LC3 conversion, an essential step for autophagy formation Cell Death Differ 2007 14 2 230 239 10.1038/sj.cdd.4401984 16794605

[B35] Wang AL Boulton ME Dunn WA Jr Rao HV Cai J Lukas TJ Neufeld AH Using LC3 to monitor autophagy flux in the retinal pigment epithelium Autophagy 2009 5 8 1190 1193 10.4161/auto.5.8.10087 19855195 PMC3704326

[B36] Rusten TE Stenmark H p62, an autophagy hero or culprit? Nat Cell Biol 2010 12 3 207 209 10.1038/ncb0310-207 20190829

[B37] Lamark T Kirkin V Dikic I Johansen T NBR1 and p62 as cargo receptors for selective autophagy of ubiquitinated targets Cell Cycle 2009 8 13 1986 1990 10.4161/cc.8.13.8892 19502794

[B38] Huang J Hao L Xiong N Cao X Liang Z Sun S Wang T Involvement of glyceraldehyde-3-phosphate dehydrogenase in rotenone-induced cell apoptosis: relevance to protein misfolding and aggregation Brain Res 2009 1279 1 8 19445904 10.1016/j.brainres.2009.05.011

[B39] Cooney R Baker J Brain O Danis B Pichulik T Allan P Ferguson DJ Campbell BJ Jewell D Simmons A NOD2 stimulation induces autophagy in dendritic cells influencing bacterial handling and antigen presentation Nat Med 2010 16 1 90 97 10.1038/nm.2069 19966812

[B40] Berg TO Fengsrud M Stromhaug PE Berg T Seglen PO Isolation and characterization of rat liver amphisomes. Evidence for fusion of autophagosomes with both early and late endosomes J Biol Chem 1998 273 34 21883 21892 10.1074/jbc.273.34.21883 9705327

[B41] Kaiser CA Schekman R Distinct sets of SEC genes govern transport vesicle formation and fusion early in the secretory pathway Cell 1990 61 4 723 733 10.1016/0092-8674(90)90483-U 2188733

[B42] Wang ZF Li HL Li XC Zhang Q Tian Q Wang Q Xu H Wang JZ Effects of endogenous beta-amyloid overproduction on tau phosphorylation in cell culture J Neurochem 2006 98 4 1167 1175 10.1111/j.1471-4159.2006.03956.x 16762022

[B43] Liu C Gao Y Barrett J Hu B Autophagy and protein aggregation after brain ischemia J Neurochem 2010 115 1 68 78 10.1111/j.1471-4159.2010.06905.x 20633207 PMC3518272

[B44] Gonzalez-Polo RA Niso-Santano M Ortiz-Ortiz MA Gomez-Martin A Moran JM Garcia-Rubio L Francisco-Morcillo J Zaragoza C Soler G Fuentes JM Inhibition of paraquat-induced autophagy accelerates the apoptotic cell death in neuroblastoma SH-SY5Y cells Toxicol Sci 2007 97 2 448 458 10.1093/toxsci/kfm040 17341480

[B45] Trojanowski JQ Lee VM "Fatal attractions" of proteins. A comprehensive hypothetical mechanism underlying Alzheimer's disease and other neurodegenerative disorders Ann N Y Acad Sci 2000 924 62 67 11193803 10.1111/j.1749-6632.2000.tb05561.x

[B46] Xue L Fletcher GC Tolkovsky AM Autophagy is activated by apoptotic signalling in sympathetic neurons: an alternative mechanism of death execution Mol Cell Neurosci 1999 14 3 180 198 10.1006/mcne.1999.0780 10576889

[B47] Canu N Tufi R Serafino AL Amadoro G Ciotti MT Calissano P Role of the autophagic-lysosomal system on low potassium-induced apoptosis in cultured cerebellar granule cells J Neurochem 2005 92 5 1228 1242 10.1111/j.1471-4159.2004.02956.x 15715672

[B48] Takacs-Vellai K Bayci A Vellai T Autophagy in neuronal cell loss: a road to death Bioessays 2006 28 11 1126 1131 10.1002/bies.20489 17041904

[B49] Hara T Nakamura K Matsui M Yamamoto A Nakahara Y Suzuki-Migishima R Yokoyama M Mishima K Saito I Okano H Suppression of basal autophagy in neural cells causes neurodegenerative disease in mice Nature 2006 441 7095 885 889 10.1038/nature04724 16625204

[B50] Komatsu M Waguri S Chiba T Murata S Iwata J Tanida I Ueno T Koike M Uchiyama Y Kominami E Loss of autophagy in the central nervous system causes neurodegeneration in mice Nature 2006 441 7095 880 884 10.1038/nature04723 16625205

[B51] Komatsu M Waguri S Koike M Sou YS Ueno T Hara T Mizushima N Iwata J Ezaki J Murata S Homeostatic levels of p62 control cytoplasmic inclusion body formation in autophagy-deficient mice Cell 2007 131 6 1149 1163 10.1016/j.cell.2007.10.035 18083104

[B52] Klionsky DJ Neurodegeneration: good riddance to bad rubbish Nature 2006 441 7095 819 820 10.1038/441819a 16778876

[B53] Anglade P Vyas S Javoy-Agid F Herrero MT Michel PP Marquez J Mouatt-Prigent A Ruberg M Hirsch EC Agid Y Apoptosis and autophagy in nigral neurons of patients with Parkinson's disease Histol Histopathol 1997 12 1 25 31 9046040

[B54] Komatsu M Wang QJ Holstein GR Friedrich VL Jr Iwata J Kominami E Chait BT Tanaka K Yue Z Essential role for autophagy protein Atg7 in the maintenance of axonal homeostasis and the prevention of axonal degeneration Proc Natl Acad Sci U S A 2007 104 36 14489 14494 10.1073/pnas.0701311104 17726112 PMC1964831

